# Psychosis spectrum features, neurocognition and functioning in a longitudinal study of youth with 22q11.2 deletion syndrome

**DOI:** 10.1017/S0033291723000259

**Published:** 2023-10

**Authors:** Raquel E. Gur, Donna M. McDonald-McGinn, Tyler M. Moore, R. Sean Gallagher, Emily McClellan, Lauren White, Kosha Ruparel, Noah Hillman, T. Blaine Crowley, Daniel E. McGinn, Elaine Zackai, Beverly S. Emanuel, Monica E. Calkins, David R. Roalf, Ruben C. Gur

**Affiliations:** 1 Brain Behavior Laboratory, Department of Psychiatry, Perelman School of Medicine, University of Pennsylvania, Philadelphia, Pennsylvania, USA; 2 Department of Child and Adolescent Psychiatry and Behavioral Sciences, Lifespan Brain Institute, Children's Hospital of Philadelphia and Penn Medicine, Philadelphia, Pennsylvania, USA; 3 22q and You Center, and Division of Human Genetics, Children's Hospital of Philadelphia, and Department of Pediatrics, Perelman School of Medicine, University of Pennsylvania, Philadelphia, Pennsylvania, USA

**Keywords:** 22q11.2 deletion syndrome, developmental trajectories, level of functioning, neurocognition, psychosis spectrum

## Abstract

**Background:**

Neuropsychiatric disorders are common in 22q11.2 Deletion Syndrome (22q11DS) with about 25% of affected individuals developing schizophrenia spectrum disorders by young adulthood. Longitudinal evaluation of psychosis spectrum features and neurocognition can establish developmental trajectories and impact on functional outcome.

**Methods:**

157 youth with 22q11DS were assessed longitudinally for psychopathology focusing on psychosis spectrum symptoms, neurocognitive performance and global functioning. We contrasted the pattern of positive and negative psychosis spectrum symptoms and neurocognitive performance differentiating those with more prominent Psychosis Spectrum symptoms (PS+) to those without prominent psychosis symptoms (PS−).

**Results:**

We identified differences in the trajectories of psychosis symptoms and neurocognitive performance between the groups. The PS+ group showed age associated increase in symptom severity, especially negative symptoms and general nonspecific symptoms. Correspondingly, their level of functioning was worse and deteriorated more steeply than the PS− group. Neurocognitive performance was generally comparable in PS+ and PS− groups and demonstrated a similar age-related trajectory. However, worsening executive functioning distinguished the PS+ group from PS− counterparts. Notably, of the three executive function measures examined, only working memory showed a significant difference between the groups in rate of change. Finally, structural equation modeling showed that neurocognitive decline drove the clinical change.

**Conclusions:**

Youth with 22q11DS and more prominent psychosis features show worsening of symptoms and functional decline driven by neurocognitive decline, most related to executive functions and specifically working memory. The results underscore the importance of working memory in the developmental progression of psychosis.

## Introduction

The 22q11.2 Deletion Syndrome (22q11DS) is heterogeneous in presentation, impacting multiple body systems, including the brain (McDonald-McGinn et al., 2015). Affected individuals may manifest a range of neuropsychiatric disorders including intellectual disability, anxiety, attention deficit hyperactivity (ADHD), autism spectrum and, in adolescence and early adulthood, schizophrenia spectrum disorders. The high prevalence of psychosis in 22q11DS (~25%) and its phenotypic similarity to idiopathic schizophrenia afford a powerful heuristic for obtaining a longitudinal perspective on the emergence of psychosis. Such information is critical in establishing developmental trajectories that can advance the study of brain and behavior, elucidate the underlying genetic architecture, and contribute to care in an informative neurogenetic syndrome.

Relative to the growing cross-sectional literature on brain-behavior in 22q11DS, there are fewer reports on longitudinal studies, commonly in small samples averaging about 3 years between baseline and follow-up. Such studies in 22q11DS often examine psychosis spectrum features. Findings from 22q11DS studies that focus on psychosis suggest that emergent psychosis symptoms relate to lower baseline IQ, externalizing symptoms and impaired social behaviors (Hooper et al., 2013); lower performance on executive function domains (Maeder et al., 2016); impaired configural face processing (Zaharia et al., 2018); lower verbal learning skills and poorer family organization (Kates et al., 2015, 2019); poor premorbid adjustment and academic achievement (Radoeva, Fremont, Antshel, & Kates, 2017); childhood anxiety symptoms and impaired attention-executive functioning (Chawner et al., 2019). A recent systematic review of 22q11DS studies identified 22 reports, out of 852 published between 2015-2019, which provided longitudinal data primarily examining the association of baseline measures with psychosis spectrum features at follow-up (Jhawar et al., 2021). Findings indicate that neurobehavioral deficits in social and executive domains and the presence of comorbid psychiatric disorders, including ADHD, anxiety, and subthreshold psychosis symptoms, are associated with an increased likelihood of psychosis outcome. However, these studies did not relate the rate of change across measures, and it is unclear how the clinical and neurocognitive parameters may affect each other in relation to functioning.

Cognitive and functioning measures in 22q11DS have been related to positive and negative symptoms of psychosis. Impaired performance on set shifting, reading decoding and emotion recognition tests were associated with positive symptoms in adulthood (Antshel, Fremont, Ramanathan, & Kates, 2017), as were baseline ultra-high-risk status and poorer level of functioning (Schneider et al., 2016). Negative symptoms were related to low functioning and persistence of at-risk state or transition to psychosis (Schneider et al., 2019), and global neurocognitive performance, executive function and social cognition deficits (Weinberger et al., 2018). Insight into risk for psychosis and its early course may be gained by examining the longitudinal trajectories of positive and negative symptoms separately in relation to specific neurocognitive domains.

The International 22q11.2 Deletion Syndrome Brain Behavior Consortium (IBBC; Gur et al., 2017) provided large samples with some retrospective longitudinal data: decline in verbal IQ was steeper in those who subsequently developed psychotic illness (Vorstman et al., 2015), as was inattention at baseline (Niarchou et al., 2019). Thus, the large-scale collaborative studies with limited longitudinal analyses generally aligned with the reports on smaller single sites, but did not suggest pathways linking specific neurocognitive domains to clinical features.

We leverage a collaborative research program between the ‘22q and You Center’ of the Children's Hospital of Philadelphia (CHOP) and the Brain-Behavior Laboratory at Penn Medicine, which tracks neurodevelopmental trajectories integrating detailed clinical and neurocognitive measures related to psychosis features and functioning. We examine the pattern and course of psychosis features differentiating those with more prominent Psychosis Spectrum features (PS+) at follow-up from those without such features (PS−). Here we present results comparing the groups on the clinical and neurocognitive change during the follow-up period and test structural equation models to determine direction of effects. We hypothesized that PS+ is associated with greater rate of neurocognitive decline that drives worsening of symptoms and poorer functioning.

## Methods

### Participants

The sample included 157 individuals with 22q11DS (PS+ 98; PS− 59) and longitudinal data, out of 564 participants in our collaborative program. All had a confirmed chromosome 22q11.2 deletion by clinical fluorescence *in situ* hybridization (FISH), SNP microarray or multiplex ligation-dependent probe amplification (MLPA). Of these, 152 individuals had deletion sizing confirmed by research MLPA which defined the breakpoints: LCR22A–LCR22D (*n* = 137, 87.26%), LCR22A–LCR22B (*n* = 7, 4.46%) LCR22A–LCR22C (*n* = 5, 3.18%), LCR22B–LCR22D (*n* = 2, 1.27%), LCR22C–LCR22D (*n* = 1, 0.64%) (Jalali et al., 2008). The remaining 5 participants did not have deletion sizing but are presumed to have an LCR22A-LCR22B deletion at minimum as the FISH probes (N25 and TUPLE) are located within this region. The recruitment for the brain-behavior study was based on consecutive presentations of patients who met study criteria (Gur et al., 2014): age > 7 years, stable health, IQ > 70. Participants underwent baseline in-person evaluation and agreed to be recontacted for longitudinal studies. Notably, CHOP is a general pediatric facility, and the participants did not present for psychiatric care. Longitudinal data collection took place from October 2008 to May 2022. Table 1 presents sample characteristics by diagnostic group. Comparison between the large sample and the current longitudinal subsample indicated no baseline differences between the groups on demographic and diagnostic symptom measures.

**Table 1.** Demographics of longitudinal sample and characteristics of groups (PS+ and PS−)

	PS− (*N* = 59)	PS+ (*N* = 98)	Overall (*N* = 157)
Sex			
Female	19(32.2%)	43(43.9%)	62(39.5%)
Male	40 (67.8%)	55(56.1%)	95(60.5%)
Race			
Black/African American	2 (3.4%)	12(12.2%)	14(8.9%)
More than one race	2 (3.4%)	6(6.1%)	8(5.1%)
White	55 (93.2%)	79(80.6%)	134(85.4%)
Native American	0(0%)	1(1.0%)	1(0.6%)
Ethnicity			
Hispanic/Latino	1 (1.7%)	10(10.2%)	11(7.0%)
Non-His panic	58(98.3%)	87(88.8%)	145(92.4%)
Unknown	0(0%)	1(1.0%)	1(0.6%)
Age at intake			
Mean (s.d.)	14.6 (4.96)	14.6 (5.37)	14.6 (5.20)
Median [Min, Max]	14.4 [7.67–25.5]	13.0 [8.00–30.1]	13.3 [7.67–30.1]
Age at last follow-up			
Mean (SO)	21.4 (5.40)	20.5(6.18)	20.8(5.90)
Median [Min, Max]	20.9 [10.3–33.6)	19.3 [10.7–34.8)	20.0 [10.3–34.8)
Timespan of follow-ups			
Mean (s.d.)	6.78 (2.76)	5.93 (2.85)	6.25 (2.84)
Median [Min. Max]	7.75 [0.833–11.5]	5.96 [0.667–12.1]	6.67 [0.667–12.1]
Number of timepoints			
2	35 (59.3%)	63(64.3%)	98(62.4%)
3	15(25.4%)	17(17.3%)	32(20.4%)
4+	9(15.3%)	18(18.4%)	27(17.2%)
Disorder comorbidity	%PS+	% PS−	
Schizophr at Time 1	3.1	0.0	
Schizophr	11.3	0.0	
Psychosis	18.6	0.0	
Prodromal	90.7	49.2	
Adhd	61.9	50.8	
Anx	81.4	64.4	
Mood	40.2	11.9	
Substance	5.2	3.4	
Other	17.5	5.1	
Medications	% PS+	% PS−	
Stimulants	29.5	25.4	
Anxiolytics	19.4	6.8	
Antidepressants	43.9	27.1	
Antipsychotic	14.3	1.7	

Most longitudinal participants were seen for follow-up at CHOP at the time the research was conducted. Therefore, they were more likely to reside in the greater Philadelphia region, primarily in Pennsylvania and New Jersey. Those seen for follow up did not differ from the larger sample on demographic or symptom measures. The number of follow-up visits ranged from 1 to 6 and with the onset of the pandemic (March 2020 onward) all research assessments were conducted remotely.

Online Supplementary Table S1 summarizes the timing of assessments and whether they were done in-person or remotely. As we focused on psychosis spectrum symptoms we limited the upper age range of the current study to 35 years as psychosis symptoms associated with schizophrenia spectrum disorders are likely to present by that age in the general population. The Institutional Review Boards of the University of Pennsylvania and CHOP approved all studies. Informed consent/assent was obtained from each participant and accompanying parent or guardian for those < 18.

### Neuropsychiatric assessment

#### Clinical

Psychiatric evaluation was conducted by highly trained assessors under the supervision of faculty and included direct assessment of the individuals with 22q11DS, collateral information from parents and review of records (Tang & Gur, 2018; Tang et al., 2014, 2017; Yi et al., 2014). A computerized adaptation of the Kiddie-Schedule for Affective Disorders and Schizophrenia (K-SADS; Kaufman et al., 1997) was administered and followed by the complete Structured Interview for Prodromal Syndromes (SIPS *v.* 4.0; Miller et al., 2003), conducted blind to initial presentation, to evaluate threshold and subthreshold psychotic symptoms. A consensus conference reviewed the obtained data and assigned a Psychosis Spectrum (PS+) status, or lack thereof (PS−), based on criteria previously detailed (Tang & Gur, 2018; Tang et al., 2014, 2017). Briefly, the 19 items on the Scale of Prodromal Symptoms (SOPS) were rated according to standardized anchors on a 7-point scale (0 = absent to 6 = severe and psychotic/extreme). Only symptoms occurring in the last six months were considered in the following domains: positive (5 items), negative (6 items), disorganization (4 items) and general (4 items) non-specific symptoms subscales (Miller et al., 2003). The presence of subthreshold psychotic symptoms was based on ratings across the positive, negative and disorganization subscales (Tang et al., 2014, 2017; Weisman et al., 2017). PS+ included individuals with one or more clinically significant (⩾3) positive symptoms and those with two or more significant negative and disorganization symptoms. The PS+ group included individuals who met diagnostic criteria for schizophrenia spectrum disorders (18.5% of overall sample) and those with subthreshold symptoms who scored in the significant range (3–5) as detailed above (75.2% of the sample). Table 1 provides demographic and clinical details on the PS+ and PS− groups. Diagnosis of other psychiatric disorders was obtained from the modified K-SADS, with greater comorbidity and psychoactive medication treatment history evident in PS+ than PS− group (see Table 1. The SIPS Global Assessment of Functioning Scale (GAF) was used to assign a consensus global functioning rating as part of the evaluation.

#### Neurocognitive

The Penn computerized neurocognitive battery (CNB) examined performance on several domains and has been applied in studies of 22q11DS (Gur et al., 2014, 2021; Weinberger et al., 2016; Yi et al., 2016). A detailed description of the battery is reported elsewhere (Gur et al., 2010, 2012; Moore, Reise, Gur, Hakonarson, & Gur, 2015). Briefly, the Penn CNB is a 1-h computerized battery consisting of 14 tests assessing five neurocognitive domains: Executive function – abstraction & mental-flexibility, attention and working memory; Episodic memory – verbal, facial, and spatial; Complex cognition – language reasoning, non-verbal reasoning and spatial processing; Social cognition – emotion identification, emotion differentiation and age differentiation; Sensorimotor speed – motor speed and sensorimotor speed. Each test provides measures of both accuracy (number of correct responses) and speed (median time for correct responses) except Sensorimotor processing tests that provide only the speed measure. Efficiency score is calculated by averaging the accuracy and speed scores of each test, where speed is scaled such that higher values indicate faster performance.

### Procedures

Study procedures typically occurred on the same day, or were split up over two sessions to accommodate the participant's schedule or minimize burden. All baseline assessments were conducted in-person, as were most follow-up pre-pandemic visits (online Supplement Table S1). The remote testing procedures, including the virtual clinical interview, CNB, and self-report scales, are nearly identical to the in-person procedures that were fully adapted for online assessments via Zoom. Participants were compensated for the assessment. Participants and their families were given the option for feedback after completion of the visit and review of data obtained.

Differences between the virtual and in-person clinical interview include the following: First, the Suicide and PTSD sections were not administered during the virtual interview. Second, during virtual assessments, certain interview sections may have required additional and more careful probing compared to an in-person interview (e.g. hygiene and motor coordination were more specifically probed since information could not easily be obtained through observation). Notably, there were minimal differences in clinical ratings or neurocognitive performance between in-person and virtual evaluations (White et al., in submission).

A case report summarizes the information obtained including proband interview, collateral interview, medical records, and school reports when available. The masters level coordinators present this report to doctoral level investigators at a weekly case conference where DSM Axis I -V diagnoses, SIPS, and Global Functioning ratings are finalized.

### Statistical analyses

The longitudinal analyses were conducted using three separate approaches: (1) generalized additive mixed modeling (Lin & Zhang, 1999; Wood, 2006); (2) ‘two-stage’ modeling in which change, or trajectory is calculated within each person before subsequent analyses using the trajectories as observed variables; (3) structural equation models examining the mediation effects across time within and among the clinical and neurocognitive variables.

#### Generalized Additive Mixed Models (GAMMs) of nonlinear trajectories

One of the best contemporary methods for describing a nonlinear function is the generalized additive model (GAM). Unlike piecewise regression (Page, 1955) GAMs allow the line segments (called ‘splines’) to actually be curve segments (nonlinear). This combination of flexible discontinuity (i.e. the ‘function’ is actually multiple functions) with nonlinearity enables GAMs to closely approximate almost any function. Because the present data are longitudinal, we used generalized additive *mixed* models (GAMMs), which can parse within-person from between-person variance to obtain unbiased standard errors. For more information about mixed models and their application in GAMMs, see McCulloch and Searle (2004) and Wood (2006), respectively. Here, GAMMs included age, diagnosis, and sex as independent variables, and six variables of interest (five neurocognitive, plus GAF) as dependent variables.

#### ‘Two-Stage’ calculation of linear slopes and downstream analyses

The ‘two-stage’ approach (e.g. Moore et al., 2017) involves: (1) calculating a slope (across time) for each person and regressing out the first time point from the slopes (to account for regression to the mean); (2) using those slopes (actually residuals) in subsequent analyses, such as a between-group comparison of mean slopes. In the present study, if a participant happened to have only two time points, the slope was still calculated using a linear model (analogous to a change score). While three points are necessary to estimate a trajectory slope and standard error, only two points are necessary to estimate a simple slope (conveniently in the same scale as trajectory slopes estimated using three points). To avoid comparisons of participants with only two timepoint to those with many repeated visits, only the first three time points were used in the two-stage analyses. Further, the inter-visit interval and interval squared were regressed out of the slopes to account for the greater possibility of extreme slopes at short intervals. The fully-adjusted slopes were then used in subsequent analyses. Mean slopes of GAF, SOPS sub-scores, and neurocognitive variables were compared among diagnostic groups (PS+/PS−) using univariate ANOVAs corrected for multiple comparisons using the false discovery rate (FDR) method. Additionally, the above models were run using linear regression while covarying for two psychopathological comorbidities emerging prior to psychosis symptom onset (ADHD and anxiety).

#### Longitudinal mediation analyses

The final analyses were aimed to understand the complex interplay between cognition and clinical symptoms over time, especially in the context of psychosis. To investigate longitudinal mediation effects from baseline (Time 1) to follow-up at Time 3 (T3), we estimated two structural equation models (Kline, 2005; Wright, 1920) in Mplus (Muthén & Muthén, 1998–2020) using maximum likelihood estimation and all paths specified (saturated model with comparative fit index = 1.0). In both models, the ‘flow’ of effects was consistent with time – i.e. variables measured at T1 predicted all later variables (T2 and T3), variables measured at T3 did not predict any previous variables, and T2 variables were both predictors and predicted. The difference between models was that the ‘Cognition -DrivesPsychosis’ model specified all effects beginning with cognition, where T1 cognition predicted all other variables (including T1 psychosis), and the ‘Psychosis Drives -Cognition’ model was specified oppositely, with T1 psychosis predicting all other variables (including T1 cognition). The goal was to determine whether there was any significant indirect effect from T1 to T3, and whether one of the two models' opposite causal configurations would produce a stronger indirect effect than the other. Additionally, as a check for whether results of the above analyses were consistent across clinical groups (PS+ and PS−), the analyses were run separately by group.

## Results

**Fig. 1.**
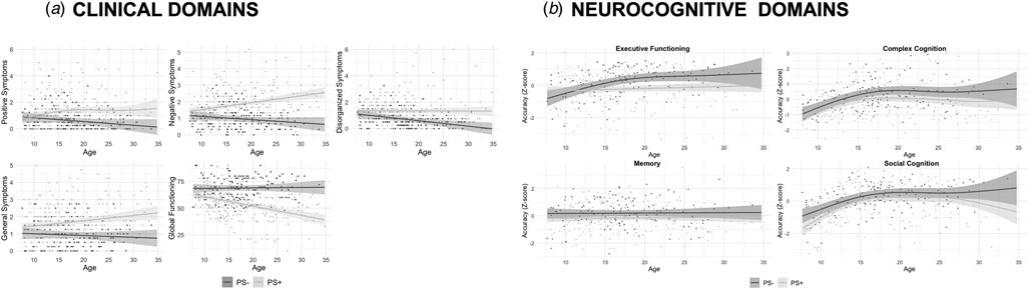
General Additive Models (GAMs) for the: a. Clinical and general level of functioning domain scores (Positive Symptoms, Negative Symptoms, Disorganized Symptoms, General Symptoms, Global adaptive functioning); b. Neurocognitive domain scores (Executive function, Memory, Complex cognition, Social cognition) for the PS+ and PS− groups across the age range.

Figure 1a shows the GAMs for the four SOPS summary scores and GAF. As can be seen, PS+ individuals are clearly distinguishable from the PS− group by increased severity of SOPS symptoms with age on all four subscales (i.e. Positive, Negative, Disorganization, and General). Correspondingly, they also show reduced level of functioning (GAF scores) across the age range.

Figure 1b shows the GAM results (± 95% CI) for the CNB efficiency score in Executive (upper left), Memory (lower left), Complex cognition (upper right), Social cognition (lower right) across the age range. As can be seen, PS+ and PS− have very comparable age-related trajectories on all domains except Executive function. For executive function, the PS− group shows improved performance with age until approximately year 20, consistent with normative expectation. The PS+ group performed comparably to the PS− group at the earliest ages (until age ~11), but showed no improvement with age, resulting in performance lower than the PS− group for most of the lifespan.

**Fig. 2.**
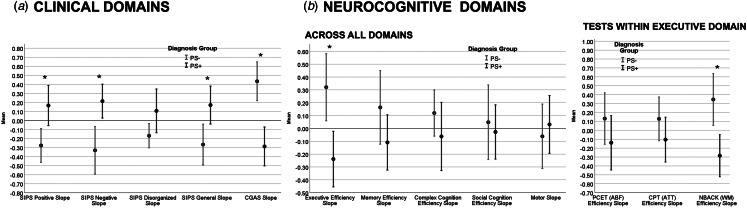
Change slopes from first to last evaluation regressing initial scoress for the PS+ and PS− group on: a. Clinical domains (Positive Symptoms, Negative Symptoms, Disorganized Symptoms, General Symptoms, Global adaptive functioning); b. Neurocognitive domain efficiency scores (Executive function, Memory, Complex cognition, Social cognition and Motor speed). The significant difference in Executive function change is further probed in the rightmost panel by showing group differences on the three tests that compose the Executive function score. PCET, Penn conditional exclusion test; ABF, Abstraction and Mental Flexibility domain; CPT, Continuous performance test; ATT, Attention domain; NBACK, N-back test; WM, Working memory.

Figure 2a shows the results of the mean slope comparisons across diagnostic category for the clinical variables. For all SOPS variables except Disorganized, PS+ group showed a significantly higher slope than the PS− group, indicating that participants with higher levels of psychosis spectrum symptoms at baseline showed faster deterioration (increasing symptoms) across time. Expectedly, GAF results indicate that PS+ participants showed a more rapid decline in functioning than PS− participants.

Figure 2b presents the mean slope comparisons across diagnostic categories for the neurocognitive variables. There is a clear pattern whereby those in the PS+ group tend to show a more rapid worsening of cognitive functioning across time than the PS− group. However, this effect was only statistically significant for the Executive domain. Table 2 shows the ANOVA results for these comparisons, as well as for those presented in Fig. 2. Note that reported *p* values are unadjusted; however, all results remain significant after FDR correction.

**Table 2.** Analysis of variance results comparing mean slopes between two diagnostic groups (PS+ and PS−)

				Mean slopes	
Variable	*F*	*p* value	Eta squared	PS−	PS+
Executive Efficiency Slope	10.59	0.001	0.077	0.321	−0.239
Memory Efficiency Slope	2.32	0.13	0.018	0.163	−0.109
Complex Cognition Efficiency Slope	1.02	0.315	0.008	0.120	−0.062
Social Cognition Efficiency Slope	0.18	0.672	0.001	0.048	−0.028
Motor Speed Slope	0.28	0.598	0.002	−0.062	0.031
GAF Slope	20	< 0.0005	0.124	0.434	−0.287
SOPS Positive Sx Slope	7.42	0.007	0.046	−0.276	0.166
SOPS Negative Sx Slope	11.57	0.001	0.07	−0.329	0.214
SOPS Disorganized Sx Slope	2.78	0.097	0.018	−0.168	0.107
SOPS General Sx Slope	7.23	0.008	0.045	−0.266	0.171

*Note*. GAF, Global Assessment of Functioning; SOPS, Scale of Prodromal Symptoms; Sx, Symptoms; eta squared values > 0.06 are commonly considered a medium effect size; *p* values are unadjusted, and all significant *p* values remain significant when FDR-adjusted.

Since change in Executive functions differed significantly between the groups, we examined further which of the executive tests (i.e. abstraction & mental-flexibility, attention and working memory) showed the most pronounced effect. As seen in the rightmost panel of Fig. 2b, only working memory (N-back task) showed a significant effect, with about equal change in abstraction & mental flexibility and attention in both groups.

When covarying for ADHD and anxiety, group differences in positive and general symptom change became non-significant (after FDR-correction, while effects for GAF, negative symptoms, and executive efficiency remained significant (corrected *p* < 0.01 for all). When further examining the three individual executive tasks while covarying for ADHD and anxiety, the effect for working memory remained significant after FDR-correction (corrected *p* = 0.004), while abstraction and attention remained non-significant.

**Fig. 3.**
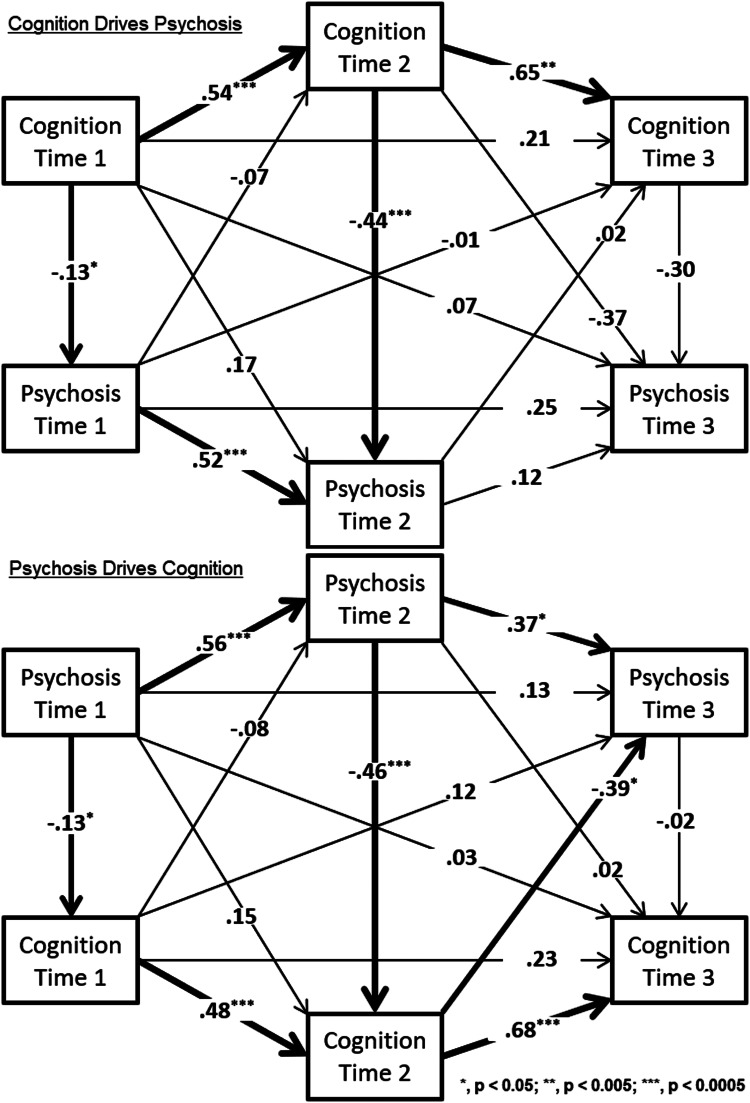
Results of the two Structural Equation Models of Longitudinal Mediation among Neurocognitive and Clinical Measure.

Figure 3 shows the results of applying structural equation modeling to evaluate mediation effects of increasing symptoms related to changes in neurocognitive function. The difference between these models comes down to which variables are residualized (and when) in the process, where the arrows from one variable to the next indicate regression models with the variable being ‘pointed at’ as the dependent variable (DV). That DV, in turn, ‘points at’ another variable (acting as an independent variable, IV), but at that point, it has been transformed to residuals resulting from the first model. For example, in the top panel of Fig. 3, ‘Psychosis Time 1’ is acting as both a DV (being predicted by ‘Cognition Time 1’) and an IV (predicting ‘Psychosis Time 2’, among other outcomes). As a DV, it is raw, untransformed data, but as an IV (acting on ‘Psychosis Time 2’), it is the residuals resulting from the model in which it was predicted by ‘Cognition Time 1’.

The top model (‘Cognition-Drives-Psychosis’) showed significant total (*β* = −0.34, *p* = 0.013) and indirect (*β* = −0.41, *p* = 0.001) paths from T1 to T3, while the model in the lower panel (‘Psychosis-Drives-Cognition’) showed neither a significant total (*β* = −0.11, *p* < 0.327) nor indirect (*β* = −0.14, *p* < 0.160) path from T1 to T3. This may be surprising given that the top model had fewer significant paths (five) than the lower model (seven), but the significance of the total and indirect effects can be substantially weakened by very small coefficients along a path. For example, in the bottom panel, three of the five final pathways to ‘Cognition Time 3’ are very small (−0.02, 0.02, and 0.03); by contrast, of the five final pathways to ‘Psychosis Time 3’ in the top panel, only one is very small (0.07). Thus, while the bottom panel contained a higher count of significant effects, the top panel was the only one to show significant total and indirect effects when all paths were considered.

Finally, the above SEMs were run separately by group (PS+ and PS−), and results were mostly consistent: the indirect effect was significant in the ‘Cognition-Drives-Psychosis’ model but not in the ‘Psychosis-Drives-Cognition’ model. The caveat is that the ‘Cognition-Drives- Psychosis’ model showed a significant indirect effect (*β* = −0.41, *p* = 0.006) only for the PS+ group, not for the PS− group. The absence of the significant indirect effect likely relates to the smaller sample and lower range of psychosis symptoms.

## Discussion

In this large comprehensively characterized longitudinal sample of individuals with 22q11.2DS we identified differences in the trajectories of psychosis spectrum symptoms and neurocognitive performance between individuals with psychosis spectrum (PS+) and those without such symptoms (PS−) at follow-up. The PS+ group showed age associated increase in symptom severity, especially for negative and general symptoms. Correspondingly, their global functioning (GAF scores) was lower and deteriorated more steeply. These effects were established by examining change scores or slopes from intake at first evaluation to second or third follow-up. These change scores were significantly higher for positive, negative and general SOPS scores in the PS+ compared to the PS− group, and correspondingly their GAF change scores indicated greater worsening rate. Notably, the PS+ group had more psychiatric comorbidities and more individuals treated with psychoactive medications than in the PS− group. Thus, the research results are validated by the clinical status that is assessed and treated independently from the research procedures. This pattern is consistent with the literature indicating greater comorbidity and subthreshold psychosis symptoms associated with more severe outcome (Chawner et al., 2019; Jhawar et al., 2021; Radoeva et al., 2017; Schneider et al., 2016, 2019). Covarying for earlier occurring ADHD and anxiety disorders suggest that comorbidities may account for the group difference that emerged in positive symptoms change scores, but did not explain the effects for negative symptoms, cognitive executive efficiency and GAF.

Neurocognitive performance was generally comparable in PS+ and PS− groups and showed a similar longitudinal trajectory. However, when specific neurocognitive domains were examined, we found that a steeper rate of decline in executive functioning distinguished the PS+ group from PS− counterparts. This finding remained significant after covarying for earlier history of ADHD and anxiety. The finding that deficits in executive functions are important and related to poorer outcome is consistent with previous studies (Antshel et al., 2017; Chawner et al., 2019; Jhawar et al., 2021; Maeder et al., 2016; Weinberger et al., 2018). However, our longitudinal analysis indicated that of the three executive functions measured (i.e. abstraction & mental flexibility, working memory, attention), only working memory showed a significant difference between the groups in rate of change. Although working memory deficits have long been implicated in idiopathic psychosis (Braun et al., 2021; Dienel & Lewis, 2019), the specificity of the longitudinal unfolding of working memory is notable, as cross-sectional findings often link psychosis to impairments in several executive functions (White et al., 2017). Thus, the current pattern of results could be more specific to psychosis in 22q11.2DS. However, five years after an initial psychotic episode in a sample without rare CNVs, González-Ortega et al. (2013) found that many core executive functions improved over time, with the exception of working memory, which remained impaired. Thus, specific decline in the ability to mentally maintain goal-related information, essential for daily functioning (Diamond, 2013), may be integrally implicated in the trajectory of psychosis in individuals with 22q11.2DS. This finding suggests a target for potential intervention, since working memory is a domain that can be improved by training (see recent meta-analysis, Ludyga, Held, Rappelt, Donath, & Klatt, 2022) and perhaps medication (Abi-Dargham *et al*., 2022).

As multiple clinical symptoms and cognitive deficits underlie schizophrenia spectrum disorders, it is important to disentangle how the longitudinal unfoldings of these domains are implicated in the emergence of psychosis. A critical inquiry is: do clinical symptoms beget cognitive decline or does poor cognition beget increased clinical symptoms? Findings from the structural equation modeling highlight the importance of neurocognitive performance as driving clinical change and thus providing potentially protective effect. A model positing that cognitive change precedes the trajectory of symptoms was supported by the data, whereas a model assuming the reverse was not. This finding underscores the need to evaluate and treat neurocognitive deficits in this population and provides foundation for the hypothesis that ameliorating cognitive dysfunction will also benefit psychiatric burden.

### Limitations

Our study has several limitations. Regarding the sample, we included individuals with IQ > 70 who can participate fully in the study, precluding generalization to individuals with lower IQ. Our follow-up includes individuals until their mid-thirties, and results should not be generalized to older age groups. However, the age range covers the age at risk for schizophrenia spectrum disorders in the general population although some younger participants are still at risk. Most of the study participants were white, reflecting the composition of individuals seen at the center and may not generalize to samples from other ancestries. The study did not use regular intervals for re-evaluations; these were done within a clinical care framework. Notably, psychiatric status was not driving the timing and nature of clinical evaluation and the results are unlikely biased in that regard. However, the naturalistic set up necessitated correction for evaluation intervals, which was applied. The wealth of medical data available will provide the opportunity to examine medical burden related to neuropsychiatric course. The two-stage model we have used can be criticized as it assumes linearity, which we know from the GAMMs is violated at least sometimes. Furthermore, the two-stage approach does not differentiate among individuals with consistent *v.* erratic trajectories. It can be useful to account for such variability in trajectory as is done, for example, in mixed models (including GAMMs), but the two-stage model assumes all trajectories are equally variable. Despite these weaknesses, however, the two-stage model has the advantage of being highly interpretable. The techniques necessary for proper estimation of longitudinal models (especially standard errors) are often complex, precluding commonsense interpretation of the numerical results. The ability of the GAMM used here to closely approximate a nonlinear function while parsing within- and between-person variance, is a strength. However, the estimation of multiple nonlinear splines within the GAMM function means the function cannot be described by a single slope and intercept. Multiple coefficients must be estimated, complicating their interpretation. By contrast, the two-stage model guarantees interpretable results, albeit at the costs just mentioned. Finally, we did not have power to examine deletion size effects.

## Conclusions

These limitations notwithstanding, our results fill an important gap in knowledge on the course and mediators of psychosis spectrum symptoms in young people with 22q11.2DS. They show that the presence of psychosis symptoms across subscales suggests worsening course and poorer adjustment. This worsening of symptoms is mediated in part by neurocognitive deficits, especially in executive function, and within executive function, specifically in working memory. The specificity to working memory could be leveraged in designing intervention studies, as the brain circuitry, long implicated in schizophrenia in human studies (Dienel, Schoonover, & Lewis, 2022) and animal models (Castner, Goldman-Rakic, & Williams, 2004)^,^ is relatively well characterized and could be target for both behavioral and pharmacologic intervention. As 22q11.2DS among rare CNVs shows a strong association with schizophrenia spectrum disorders, it provides a unique opportunity to examine the precursors and course of the emergence of psychosis. The information offers translational opportunities in animal models of 22q11.2 deletion, probing working memory circuitry (Tamura, Mukai, Gordon, & Gogos, 2016). Use of such models can help investigate the impact of stress and examine interventions to improve performance. The findings can also guide ascertainment strategies of establishing induced pluripotent stem cells and lymphoblastoid cell lines to advance mechanistic understanding that can potentially propel treatments (Li et al., 2021). For example, selecting cell lines from individuals with 22q11DS at tails of the distribution for psychosis (PS+) and working memory.

## Supplementary material

For supplementary material accompanying this paper visit https://doi.org/10.1017/S0033291723000259. click here to view supplementary material
