# Supplementary figures and images for: Psychosis spectrum features, neurocognition and functioning in a longitudinal study of youth with 22q11.2 deletion syndrome
Source: Psychol Med. 2023 Mar 29;53(14):6763–72. doi: 10.1017/S0033291723000259 (PMC10600823; doi:10.1017/S0033291723000259)

**Supplement Table S1.** Distribution of sample by time point and remote vs. in-person assessment


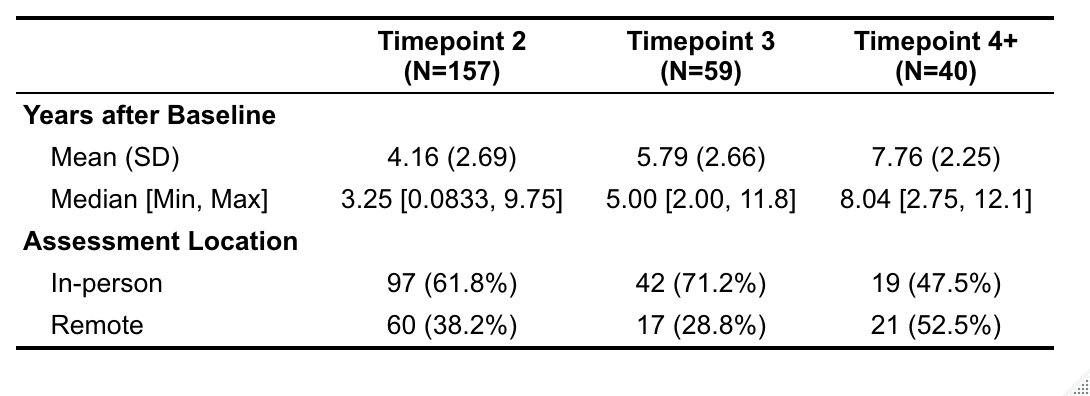

Supplement: Supplementary file 1 [file S0033291723000259sup001.docx]
